# Effectiveness of the booster dose of inactivated COVID-19 vaccine against Omicron BA.5 infection: a matched cohort study of adult close contacts

**DOI:** 10.1186/s12931-023-02542-y

**Published:** 2023-10-12

**Authors:** Ting Zeng, Yaoqin Lu, Yanji Zhao, Zihao Guo, Shengzhi Sun, Zhidong Teng, Maozai Tian, Jun Wang, Shulin Li, Xucheng Fan, Weiming Wang, Yongli Cai, Gengze Liao, Xiao Liang, Daihai He, Kai Wang, Shi Zhao

**Affiliations:** 1 School of Public Health, Xinjiang Medical University, Urumqi, 830017 China; 2 Urumqi Center for Disease Control and Prevention, Urumqi, 830026 China; 3 Department of Applied Mathematics, Hong Kong Polytechnic University, Hong Kong, 999077 China; 4 JC School of Public Health and Primary Care, Chinese University of Hong Kong, Hong Kong, 999077 China; 5 School of Public Health, Capital Medical University, Beijing, 100069 China; 6 Department of Medical Engineering and Technology, Xinjiang Medical University, Urumqi, 830017 China; 7 School of Mathematics and Statistics, Huaiyin Normal University, Huaian, 223300 China; 8 Department of Rehabilitation Sciences, Hong Kong Polytechnic University, Hong Kong, 999077 China; 9 Research Institute for Future Food, Hong Kong Polytechnic University, Hong Kong, 999077 China; 10 Centre for Health Systems and Policy Research, Chinese University of Hong Kong, Hong Kong, 999077 China

**Keywords:** COVID-19, Omicron variant, Vaccine effectiveness, Asymptomatic infection

## Abstract

**Background:**

Although COVID-19 vaccines and their booster regimens protect against symptomatic infections and severe outcomes, there is limited evidence about their protection against asymptomatic and symptomatic infections in real-world settings, particularly when considering that the majority of SARS-CoV-2 Omicron infections were asymptomatic. We aimed to assess the effectiveness of the booster dose of inactivated vaccines in mainland China, i.e., Sinopharm (BBIBP-CorV) and Sinovac (CoronaVac), against Omicron infection in an Omicron BA.5 seeded epidemic.

**Methods:**

Based on an infection-naive but highly vaccinated population in Urumqi, China, the study cohort comprised all 37,628 adults who had a contact history with individuals having SARS-CoV-2 infections, i.e., close contacts, between August 1 and September 7, 2022. To actively detect SARS-CoV-2 infections, RT-PCR tests were performed by local authorities on a daily basis for all close contacts, and a testing-positive status was considered a laboratory-confirmed outcome. The cohort of close contacts was matched at a ratio of 1:5 with the fully vaccinated (i.e., 2 doses) and booster vaccinated groups (i.e., 3 doses) according to sex, age strata, calendar date, and contact settings. Multivariate conditional logistic regression models were adopted to estimate the marginal effectiveness of the booster dose against Omicron BA.5 infection after adjusting for confounding variables. Subgroup analyses were performed to assess vaccine effectiveness (VE) in different strata of sex, age, the time lag from the last vaccine dose to exposure, and the vaccination status of the source case. Kaplan–Meier curves were employed to visualize the follow-up process and testing outcomes among different subgroups of the matched cohort.

**Findings:**

Before matching, 37,099 adult close contacts were eligible for cohort enrolment. After matching, the 2-dose and 3-dose groups included 3317 and 16,051 contacts, and the proportions with Omicron infections were 1.03% and 0.62% among contacts in the 2-dose and 3-dose groups, respectively. We estimated that the adjusted effectiveness of the inactivated booster vaccine versus 2 doses against Omicron infection was 35.5% (95% CI 2.0, 57.5). The booster dose provided a higher level of protection, with an effectiveness of 60.2% (95% CI 22.8, 79.5) for 15–180 days after vaccination, but this VE decreased to 35.0% (95% CI 2.8, 56.5) after 180 days. Evidence for the protection of the booster dose was detected among young adults aged 18–39 years, but was not detected for those aged 40 years or older.

**Interpretation:**

The receipt of the inactivated vaccine booster dose was associated with a significantly lower Omicron infection risk, and our findings confirmed the vaccine effectiveness (VE) of booster doses against Omicron BA.5 variants. Given the rapid evolution of SARS-CoV-2, we highlight the importance of continuously monitoring the protective performance of vaccines against the genetic variants of SARS-CoV-2, regardless of existing vaccine coverage.

## Introduction

Although vaccine program is an effective strategy in fighting against the COVID-19 pandemic [1–3], the current global predominance of SARS-CoV-2 Omicron variants continuously challenges vaccine-induced protection, which was recognized by the World Health Organization (WHO) as one of the major public health concerns. Evidence from recent studies also suggested that the immunity generated by vaccination may wane over time [4–10], and Omicron variants were associated with increased transmissibility and immune escape ability [11–15]. The booster dose of vaccines was used to enhance immunity levels [16, 17], and was reported to provide relatively high protection against symptomatic to severe COVID-19 outcomes, including hospitalization, need for intensive care, and death [18–24]. Most existing estimates of vaccine effectiveness (VE) against Omicron infections have focused on various mRNA vaccines, including mRNA-1273 and BNT162b2, or adenovirus vector vaccines, such as ChAdOx1-nCoV-19 [24–28]. The COVID-19 vaccines received by almost all vaccinees in mainland China were Sinopharm (BBIBP-CorV) and Sinovac (CoronaVac) inactivated COVID-19 vaccines, mainly BBIBP-CorV in Urumqi city (the city where the cohort was recruited in our study). Although the efficacy of inactivated vaccines was assessed in phase III clinical trials [17, 29], the real-world evidence of the effectiveness of inactivated vaccines remains largely unassessed [30, 31], especially considering the challenges caused by genetic variants of SARS-CoV-2, and immunity waning after vaccination.

Considering that the majority of Omicron infections may not progress to pneumonia and some of them are subclinical [32, 33], real-world evidence of VE in preventing mild and asymptomatic Omicron infections is generally lacking, but it is important for developing herd immunity. The evaluation of VE against asymptomatic and mild infections is potentially challenging because infections without identifiable symptoms were less likely to be ascertained. As such, VE estimates under common study designs, including test-negative designs, could bias toward more severe clinical conditions or subgroups of populations with relatively high test-seeking behaviors [34], which may fail to be a fair representation of all infections. To our knowledge, there is only one study that assessed the effectiveness of inactivated vaccines against (asymptomatic and symptomatic) Omicron BA.2 infection in Hong Kong by using a cohort design, and the cohort was collected from participants randomly selected from the general population [35]. However, the contact tracing information was uninvestigated in their study, such that the determinants that contribute to secondary transmission, e.g., contact settings or the vaccination status of source cases, and then the downstream infection of close contacts remained unadjusted for or studied. In addition, the ongoing (as of December 2022) COVID-19 pandemic was dominated by Omicron BA.5 and its genetic sublineages [36], which have replaced Omicron BA.2 globally since the middle of 2022; thus, updating the VE against the (most recent) circulation SARS-CoV-2 variants may inform the risk assessment of current COVID-19 situations.

In this study, we assessed the effectiveness of the booster dose of inactivated vaccines against asymptomatic and symptomatic Omicron BA.5 infections in a well-traced cohort including all documented adult COVID-19 close contacts from August 1 to September 7, 2022. This cohort was collected from an infection-naive population with relatively high vaccination coverage in Urumqi, the capital and largest city in the Xinjiang Uygur Autonomous Region, China.

## Methods

This was a retrospective cohort study including all adult close contacts of COVID-19 in Urumqi from August 1 to September 7, 2022. The study followed the Strengthening Reporting of Observational Studies in Epidemiology (STROBE) reporting guidelines. The collection of specimens, epidemiological and clinical data for SARS-CoV-2 infected individuals and their close contacts is part of a continuing public health investigation of COVID-19 outbreaks, ruled in the Protocol on the Prevention and Control of COVID-19 by the National Health Commission of the People’s Republic of China, which was exempt from ethical approval (i.e., institutional review board assessment). This study was approved by the institutional ethics committee of Xinjiang Medical University. Individual verbal consent was obtained when collecting personal information and human samples by governmental healthcare professionals in the field. All study data were completely anonymized. This study used secondary data without personal identity or human sample provided by the Urumqi Center for Disease Control and Prevention.

### Study setting

Mainland China implemented the “zero COVID-19” policy from 2020 to October 2022 (after the end of our study period), and thus, no large-scale COVID-19 outbreak occurred in the context of “zero COVID-19” control measures in Urumqi before August 2022, which means that the population, with a size of 3.8 million, was (largely) infection-naive. The COVID-19 vaccines received by almost all vaccinees in mainland China were Sinopharm vaccines (BBIBP-CorV) and Sinovac vaccines (CoronaVac), which were inactivated COVID-19 vaccines developed and administered under the supervision of Chinese authorities. Among all vaccinated subjects included in this study, BBIBP-CorV and CoronaVac were administered, and the majority were BBIBP-CorV. Since both BBIBP-CorV and CoronaVac were inactivated vaccines with similar contents, vaccine types were not further compared in this study. By the end of July 2022, before the start of the study period, the coverage of 2-dose and booster inactivated vaccines was 90% and > 72% [37], respectively, for the general population of mainland China, which was similar to that in Urumqi city. Most of the non-vaccinees (i.e., those who received 0 dose) in mainland China were those with existing conditions making them unsuitable for receiving vaccines due to medical concerns.

During the period from August 1 to September 7, 2022, the first group of COVID-19 cases in Urumqi, which is an epicenter of the outbreak, was detected. The outbreak was seeded by Omicron BA.5.2 variants (classified using PANGO lineage designation [38]), and the confirmation of these genetic variants was conducted through whole-genome sequencing of 11 randomly selected COVID-19 cases in the initial days of the outbreak. The outbreak in the Xinjiang Uygur Autonomous Region, China, started on August 7 and reached its peak on August 13. According to the “zero COVID-19” policy, a series of intensive control measures were then swiftly implemented by the local government on August 10, including city-wide lockdown, travel ban, mass case detection, symptom-based surveillance, contact tracing, case isolation and contact quarantine. Since the start of the outbreak, mandatory reverse transcription polymerase chain reaction (RT-PCR) tests were administered by the local authority on a daily basis for all citizens in Urumqi (city-wide mass testing). Test-positive individuals and his/her close contacts were immediately quarantined.

All individuals who had an epidemiological link to a laboratory-confirmed COVID-19 case were classified as close contacts of COVID-19. Information on exposure history was collected and documented through interviews with individuals with confirmed COVID-19 cases as well as their digital records of travel history through an online platform (i.e., China’s COVID-tracking QR code downloaded on individual mobile phones). As a major part of the contact tracing program conducted by the city-level Centers for Disease Control and Prevention, the contact history of each individual who was suspected to have exposure risks was linked to COVID-19 cases on a pairwise basis. The epidemiological link was identified for individuals who had unprotected contact [e.g., without sufficient personal protective equipment (PPE)] with a COVID-19 case within 4 days before his or her test-positive date, because a considerable amount of transmission could occur at an early stage after infection.

With large efforts to actively detect SARS-CoV-2 infections in Urumqi, as well as other places in mainland China (before November 2022), mandatory reverse transcription polymerase chain reaction (RT-PCR) tests were administered by the local authority on a daily basis for all close contacts. SARS-CoV-2 infections were laboratory-confirmed by performing RT-PCR tests (cycle threshold [Ct] value < 40) on specimens collected from nasopharyngeal or oropharyngeal swabs.

### Study design, participants, and variables

**Fig. 1:**
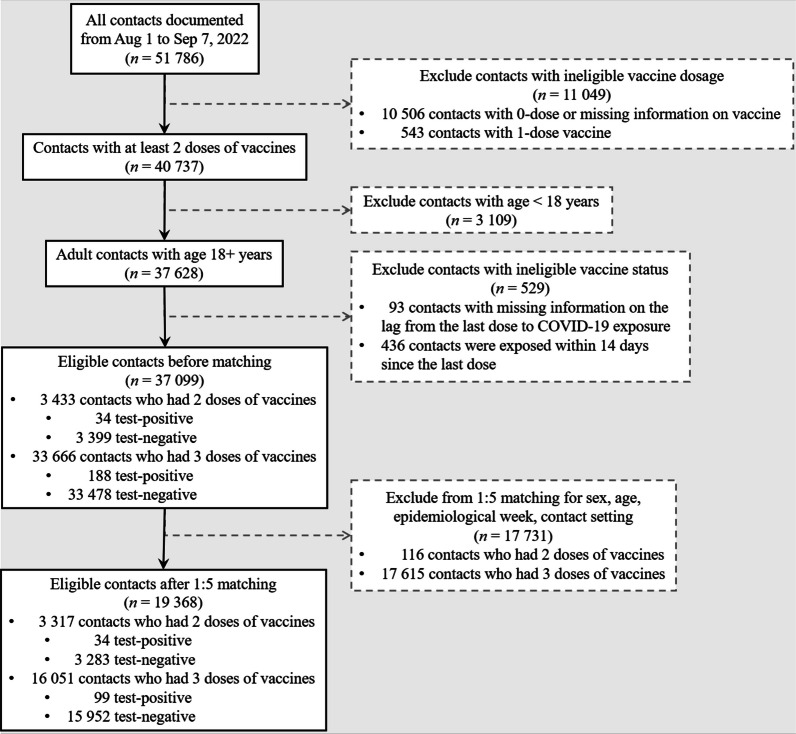
Flowchart of samples selection, and subsequent propensity score matching before statistical analyses

This was a matched cohort study including all adult close contacts of COVID-19 between August 1 and September 7, 2022, in Urumqi, China. For participant selection before matching, we excluded contacts who had received fewer than 2 doses of vaccines because we aimed to study booster vaccination versus 2-dose vaccination among adults. Note that only a small proportion (1.0%) of participants were partially vaccinated (i.e., received 1 dose). Those contacts who had missing information on the date of the last vaccine dose (before exposure) were excluded. Those exposed within 14 days since the last dose were also excluded [31], which was to account for the time lag for vaccines to develop protective effects within human hosts [39]. The participants’ selection procedures are visualized in the flowchart shown in Fig. 1.

For the eligible contacts, we extracted individual-level information, including the age and sex of both the contacts and their linked source cases, contact settings (i.e., household, community, workplace, and unknown settings), timeline-list data of vaccination and exposure history, vaccination status of source cases, and RT-PCR test result for SARS-CoV-2 infection. The vaccination status of close contacts was considered the variable of interest, and we considered 2-dose vaccination as the reference level against booster vaccination. For the outcome, we considered RT-PCR test-positive status for SARS-CoV-2 infection as the primary outcome variable for both asymptomatic and symptomatic infections. As most of the Omicron infections were asymptomatic or mildly symptomatic [32], we also observed that < 10% of adult infections in our cohort were symptomatic.

### Propensity score matching

The propensity score was estimated by a multivariate logistic regression model. In this study, close contacts who received (only) 2 doses of vaccine were matched to close contacts who received the booster (i.e., third) dose of vaccine at a ratio of 1:5 using the nearest-neighbor approach with discard, where participants were matched for sex, the age of both index (source) cases and close contacts, the calendar date of contact (in the form of the epidemiological week of 2022), and contact setting strata. These variables were chosen based on possible or known associations with transmission risks or contact patterns to reduce the likelihood of selection bias in vaccination status and transmission risks, and facilitated the comparison between the testing outcomes of SARS-CoV-2 based on vaccination status.

For each variable, the after-matching standardized mean differences (SMD) between 2-dose and 3-dose vaccinees were calculated, and SMD < 0.1 was considered a satisfactory balance of the baseline conditions between the two cohorts [40, 41]. The matched cohort was used to assess the vaccine effectiveness.

### Statistical analyses

We stratified the cohort by vaccination status and testing outcome for SARS-CoV-2 infections. The characteristics of the eligible (i.e., before-match) and after-matching cohorts were described with the use of frequency distributions and measures of central tendency.

Using the matched cohort, multivariate conditional logistic regression models were adopted to explore the association between vaccination status and SARS-CoV-2 infection risk among close contacts with COVID-19 in terms of the odds ratio (OR). The vaccine effectiveness (VE) was calculated based on the OR, such that VE = (1 − OR) × 100% when OR < 1; or VE = − (1 − 1/OR) × 100% when OR > 1 [42–44]. We controlled for potential confounding variables, including the sex and age of both source cases and contacts, the epidemiological week of contact history, the vaccination status of source cases, and contact settings. We assessed the statistical uncertainty by using the 95% confidence interval (CI). Our reference group was the two-dose group, so the vaccine effectiveness we studied was marginal VE. Although survival analysis with time-varying risk can be applied to the estimation of VE in situations that proportional hazard (PH) assumption was violated [35, 45], we adopted conditional logistic regression models [46, 47], which was conservative for the retrospective data after matching.

Subgroup analyses were performed in which we assessed VE by sex (male and female), age (18–39, 49–60, and ≥ 61 years), time lag from last vaccine dose to contact with COVID-19 cases (15–180, and ≥ 181 days), and the vaccination status of source case (0–1 dose, 2, and 3 doses). For data visualization, we employed the Kaplan–Meier estimator to construct cumulative incidence curves [48]. The hazards of Omicron infection were stratified by the vaccine dose of contacts, and compared using log-rank tests for a statistically significant difference.

All data processing and matching procedures were performed in **R** statistical software (version 4.1.1) [49], and specifically, propensity score matching was conducted using the package “*MatchIt*” [50].

### Role of the funding sources

The funding sources had no role in the design, conduct, and reporting of the study or in the decision to submit the manuscript for publication.

## Results

From August 1 to September 7, 2022, there were 19,368 individuals in close contact with COVID-19 after strict screening criteria, including 3317 and 16,051 individuals in the 2-dose and 3-dose groups, respectively (Fig. 1). The covariates in Table 1 were balanced between the 2 cohorts after matching (with SMD < 0.1). Similar to the two-dose group, the median age in the three-dose group was 36 years old (IQR: [28.0, 51.0]), with 54.0% female cases and 58.0% young adults (aged 18–39 years). We observed that over 50% of cases among close contact were detected in the 32nd epidemiological week, which was consistent with the peak date of the epidemic wave on August 10, 2022. Although most contact settings were unclassified, the distribution of cases among known contact settings, including household, community, and workplace, was approximately even in both the 2-dose and the 3-dose group.

**Table 1:** Baseline characteristics of the eligible (before-match) and matched cohorts of adult close contacts of COVID-19 who received 2-dose and 3-dose vaccine

Characteristics	Eligible cohorts			Matched cohorts		
	2 doses, *n* (column %)	3 doses, *n* (column %)	SMD	2 doses, *n* (column %)	3 doses, *n* (column %)	SMD
Total	3433 (100%)	33,666 (100%)	NA	3317 (100%)	16,051 (100%)	NA
Sex						
Male	1571 (45.8%)	16,061 (47.7%)	0.039	1555 (46.9%)	7388 (46.0%)	0.017
Female	1862 (54.2%)	17,605 (52.3%)		1762 (53.1%)	8663 (54.0%)	
Age group						
Young adult: 18–39 yr	1868 (54.4%)	16,241 (48.2%)	0.483	1868 (56.3%)	9313 (58.0%)	0.071
Middle-age adult: 40–59 yr	851 (24.8%)	14,665 (43.6%)		851 (25.7%)	4271 (26.6%)	
Old-age adult: 60+ yr	714 (20.8%)	2760 (8.2%)		598 (18.0%)	2467 (15.4%)	
Median age, yr [IQR]	37.0 [28.0, 55.0]	40.0 [31.0, 51.0]	0.074	36.0 [28.0, 53.0]	36.0 [28.0, 51.0]	0.070
Epidemiological week of 2022						
wk 31: Jul 31–Aug 6	775 (22.6%)	7230 (21.5%)	0.077	749 (22.6%)	3644 (22.7%)	0.047
wk 32: Aug 7–Aug 13	1683 (49.0%)	17,510 (52.0%)		1670 (50.3%)	8261 (51.5%)	
wk 33: Aug 14–Aug 20	470 (13.7%)	3992 (11.9%)		406 (12.2%)	1970 (12.3%)	
wk 34: Aug 21–Aug 27	306 (8.9%)	2835 (8.4%)		293 (8.8%)	1236 (7.7%)	
wk 35: Aug 28–Sep 3	127 (3.7%)	1257 (3.7%)		127 (3.8%)	562 (3.5%)	
wk 36: Sep 4–Sep 10	72 (2.1%)	842 (2.5%)		72 (2.2%)	378 (2.4%)	
Contact setting						
Household	101 (2.9%)	874 (2.6%)	0.069	94 (2.8%)	373 (2.3%)	0.036
Community	131 (3.8%)	1198 (3.6%)		119 (3.6%)	551 (3.4%)	
Workplace	94 (2.7%)	1317 (3.9%)		94 (2.8%)	497 (3.1%)	
Unknown settings	3107 (90.5%)	30,277 (89.9%)		3010 (90.7%)	14,630 (91.1%)	
Age group of source case						
Minor: < 18 yr	230 (6.7%)	1784 (5.3%)	0.107	210 (6.3%)	871 (5.4%)	0.070
Young adult: 18–39 yr	1428 (41.6%)	15,193 (45.1%)		1428 (43.1%)	7223 (45.0%)	
Middle-age adult: 40–59 yr	1511 (44.0%)	14,733 (43.8%)		1469 (44.3%)	6995 (43.6%)	
Old-age adult: 60+ yr	264 (7.7%)	1956 (5.8%)		210 (6.3%)	962 (6.0%)	
Vaccine status of source case						
0–1 dose	267 (7.8%)	2473 (7.3%)	0.054	261 (7.9%)	1130 (7.0%)	0.051
2 doses	457 (13.3%)	3928 (11.7%)		407 (12.3%)	1881 (11.7%)	
3 doses	2709 (78.9%)	27,265 (81.0%)		2649 (79.9%)	13,040 (81.2%)	

**Fig. 2:**
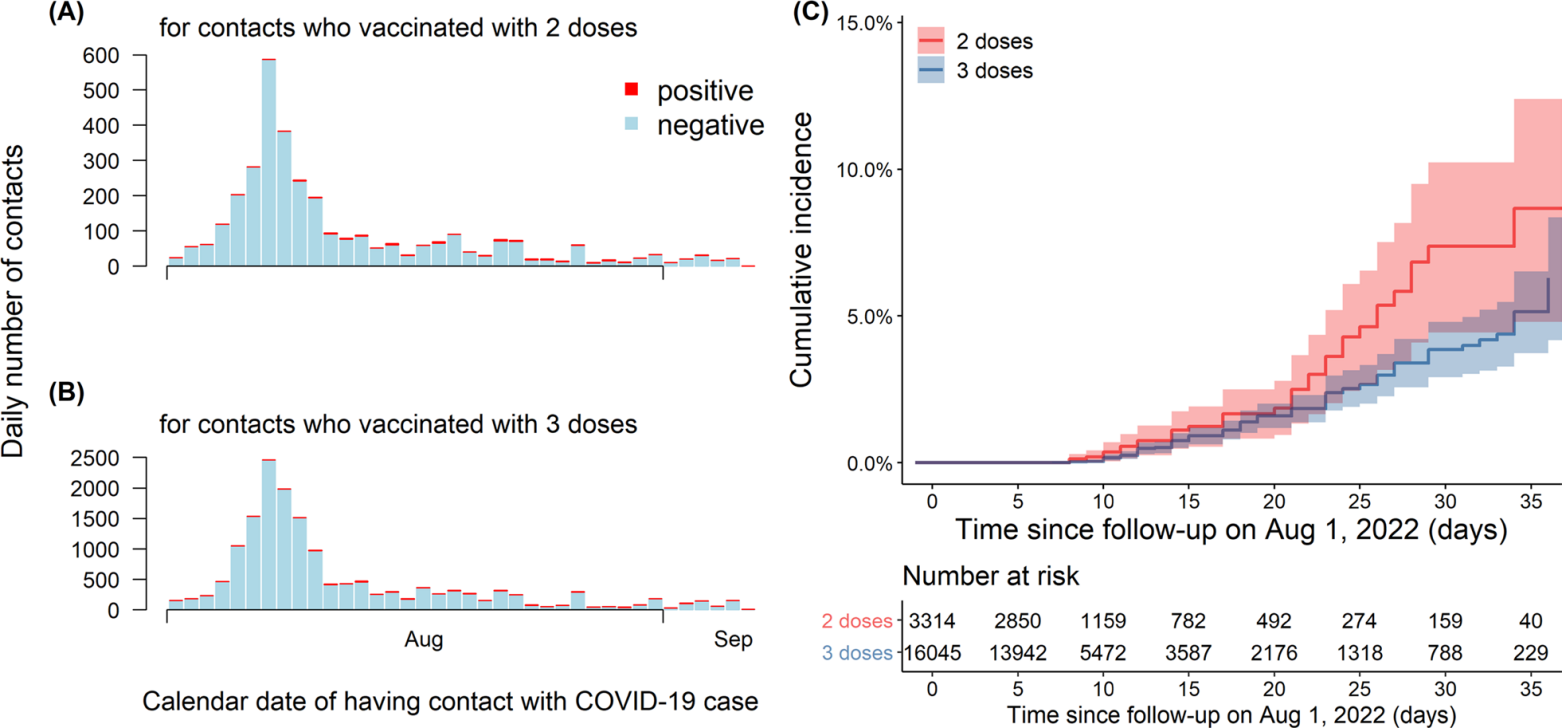
The time series of close contacts by the calendar date of having contact with a source case, stratified by RT-PCR testing status (test-positive in red, and test-negative in cyan) for SARS-CoV-2 infection, and vaccine doses (2 doses in **A**, and 3 doses in **B**) received before having contact. **C** Showed the cumulative incidence of SARS-CoV-2 Omicron infections stratified by vaccine doses

Figure 2A, B illustrated daily reported cases in close contact with COVID-19 in the 2-dose and 3-dose groups, respectively, in which the number of cases peaked on August 7, and decreased in both groups due to the city lockdown. Figure 2C showed the cumulative incidence rates for the two- and three-dose groups, and the rate of the two-dose group exhibited a more significant upward trend with follow-up days after August 7, 2022. The median lengths of follow-up of individuals administered two and three doses of inactivated vaccine were 8 person-days (ranging from 7 to 15) and 8 person-days (ranging from 7 to 14), respectively. Table 2 showed the vaccine effectiveness of two doses and three doses of inactivated vaccine against infection by the Omicron BA.5 variants. A higher incidence rate was shown in the 2-dose group than in the 3-dose group within each stratum. The overall VE of the booster dose against Omicron BA.5 infection was 35.5% (95% CI 2.0, 57.5). Although a similar magnitude of effectiveness between males and females is shown in Table 2, the protective effect for individuals aged 18–39 years was higher, with a VE of 40.2% (95% CI 4.8, 62.5) than for other age groups. Booster vaccination was associated with significant time resistance, and a decline in effectiveness from 60.2% (15–180 days after vaccination) to 35.0% (more than 180 days after vaccination). Compared to other vaccination statuses, the effectiveness for primary cases who had received 3 doses of vaccine was relatively higher, with a VE of 39.9% (95% CI 6.7, 61.3).

**Table 2:** Summary of the effectiveness of 3-dose inactivated vaccine, versus 2-dose (reference level), against SARS-CoV-2 Omicron BA.5 infection

	Sample size, *n* (column %)				Length of follow-up (person-day), median [IQR]		Incidence rate (per 100,000 person-day)		Vaccine effectiveness, estimate (95% CI)	
	2-dose vaccinee (ref.)		3-dose vaccinee							
	Test-positive	Test-negative	Test-positive	Test-negative	2-dose	3-dose	2-dose	3-dose	Crude	Adjusted^a^
Overall	34 (100%)	3283 (100%)	99 (100%)	15,952 (100%)	8 [7, 15]	8 [7, 14]	88.51	53.67	40.1% (11.4, 59.5)	35.5% (2.0, 57.5)
Sex										
Male	16 (47.1%)	1539 (46.9%)	49 (49.5%)	7399 (46.4%)	9 [7, 17]	9 [7, 16]	85.05	54.37	35.8% (− 11.7, 63.6)	39.7% (3.2, 62.5)
Female	18 (52.9%)	1744 (53.1%)	50 (50.5%)	8643 (54.2%)	8 [7, 14]	8 [7, 13]	91.83	53.01	43.8% (3.4, 67.3)	39.9% (6.6, 61.3)
Age stratification										
Young adult: 18–39 yr	11 (32.4%)	1857 (56.6%)	51 (51.5%)	9262 (58.1%)	8 [7, 13]	8 [7, 13]	53.74	49.15	7.0% (− 44.0, 51.6)	40.2% (4.8, 62.5)
Middle-age adult: 40–59 yr	8 (23.5%)	843 (25.7%)	30 (30.3%)	4241 (26.6%)	9 [7, 17]	9 [7, 16]	76.72	58.80	25.5% (− 38.7, 65.9)	33.2% (− 8.9, 59.3)
Old-age adult: 60+ yr	15 (44.1%)	583 (17.8%)	18 (18.2%)	2449 (15.4%)	9 [7, 18]	8 [7, 15]	199.55	60.66	71.4% (43.0, 85.7)	37.8% (− 10.1, 65.2)
Lag from last vaccine to be exposed										
15–180 d	15 (44.1%)	1208 (36.8%)	15 (15.2%)	3594 (22.5%)	8 [7, 14]	8 [7, 13]	107.61	37.69	66.4% (31.0, 83.6)	60.2% (22.8, 79.5)
181+ d	19 (55.9%)	2075 (63.2%)	84 (84.8%)	12,358 (77.5%)	8 [7, 15]	9 [7, 15]	77.63	58.07	25.8% (− 18.3, 55.0)	35.0% (2.8, 56.5)
Vaccine status of source case										
0–1 dose	3 (8.8%)	258 (7.9%)	12 (12.1%)	1118 (7.0%)	14 [6, 22]	13 [6, 22]	76.22	73.91	7.7% (− 69.7, 74.1)	29.2% (− 30.8, 65.3)
2 doses	10 (29.4%)	397 (12.1%)	20 (20.2%)	1861 (11.7%)	10 [6, 23]	10 [6, 23]	172.27	74.89	57.3% (8.1, 80.2)	31.4% (− 27.5, 65.9)
3 doses	21 (61.8%)	2628 (80.0%)	67 (67.7%)	12,973 (81.3%)	8 [7, 13]	8 [7, 12]	73.24	47.35	35.4% (− 5.4, 60.5)	39.9% (6.7, 61.3)

^a^The vaccine effectiveness (VE) was estimated from multivariate conditional logistic regression model adjusted for covariables including sex, age, epidemiological week of 2022, contact setting, and vaccine status of source case

**Fig. 3:**
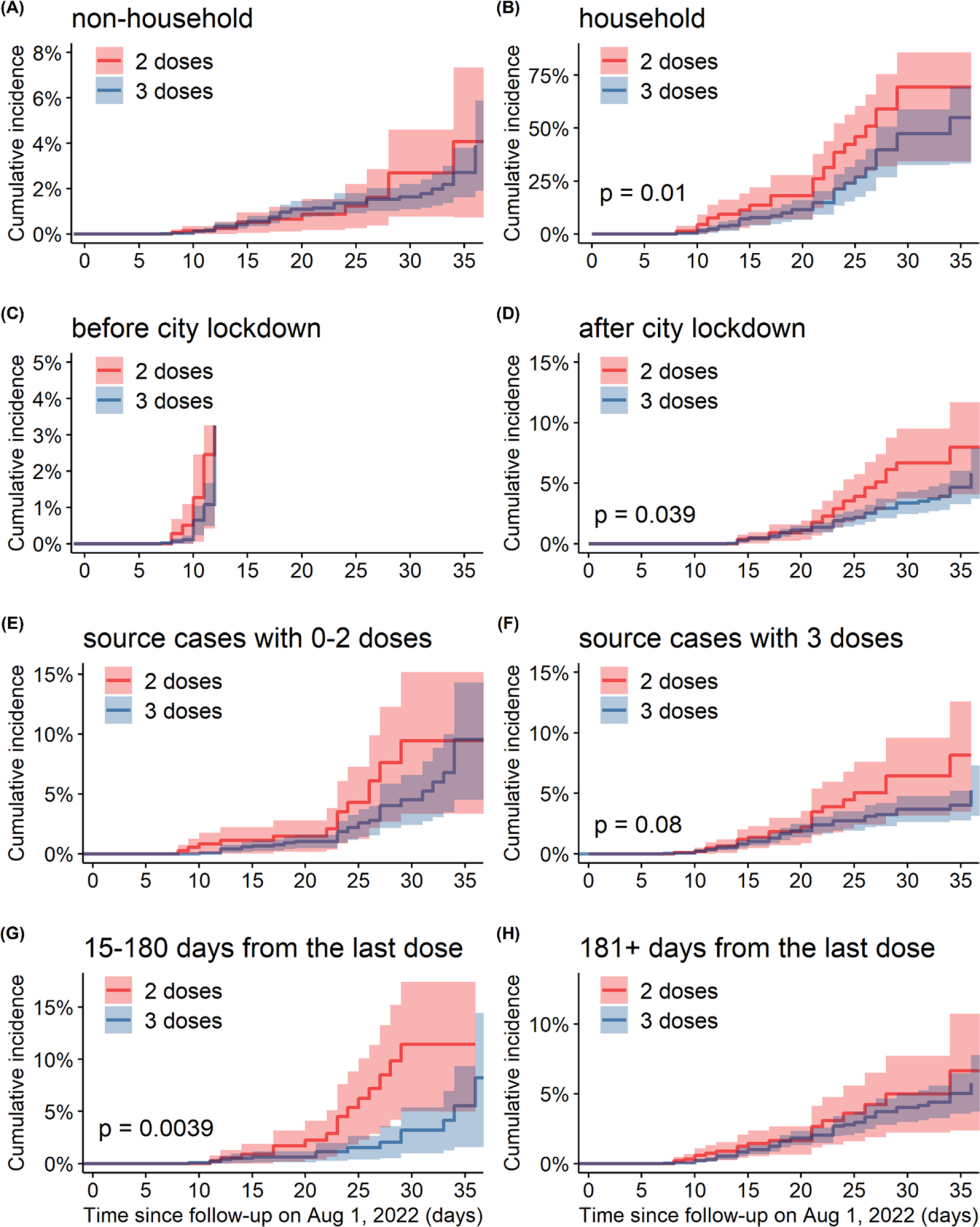
Cumulative incidence of Omicron BA.5 infections in the matched cohorts of adult close contacts of COVID-19 who received 2-dose (red curve) and 3-dose (blue curve) vaccine, stratified by contact settings (non-household versus household setting in **A** and **B**), time periods of city lockdown (before versus after city lockdown in **C** and **D**), vaccine statuses of source cases (0–2 versus 3 doses in **E** and **F**), and time from the last vaccine dose to having contact with COVID-19 cases (15–180 versus 181+ days in **G** and **H**)

The cumulative incidence of Omicron BA.5 infections stratified into various subgroups is visualized in Fig. 3. In the household subgroups (Fig. 3B), after lockdown (Fig. 3D), and 15 to 180 days after the last dose (Fig. 3G), the incidences in the two-dose vaccine group were significantly higher than those in the three-dose vaccine group (*p*-values of 0.01, 0.04, and < 0.01, respectively). The risk of infection among those in the relevant strata who received three doses of vaccine was lower than that among those who received two doses. Among other subgroups, the differences between the 2-dose and 3-dose groups were not statistically evident.

## Discussion

In 2022, Omicron variants and their genetic sub-lineages continuously posed new threats to public health globally. Compared to the ancestral strain of SARS-CoV-2 and other types of variants of concern, the Omicron variant is more likely to spread via surfaces and aerosol vectors [51]. In addition to the incubation period after Omicron infection being shortened, the majority of Omicron infections were asymptomatic or mildly symptomatic, and the risks of hospitalization and death were reported at a relatively low level [51, 52]. Therefore, researchers have been increasingly concentrating on VE against Omicron variants and its change over time. In Urumqi, the RT-PCR tests were performed for all close contacts once per week when there was no local case reported, once every 2 or 3 days when sporadic local transmission chains were reported, and once per day during the outbreak of the epidemic. As such, this study provided a real-world assessment of the effectiveness of a third dose of inactivated vaccine against Omicron BA.5 infection regardless of symptom status.

The inactivated COVID-19 vaccine protected against both asymptomatic and symptomatic SARS-CoV-2 Omicron infection, but the magnitude of protection depended on the dose of the vaccine. Based on a population-based observational study evaluating the VE of 1, 2 and 3 doses of the BNT162b2 and CoronaVac vaccines against Omicron BA.2 in Hong Kong. Similar findings to ours have been reported regarding the significant effectiveness of three doses of the BNT162b2 (50.9%) and CoronaVac (41.6%) vaccines against Omicron BA.2 infection compared to unvaccinated [35]. McMenamin et al. stated a conclusion consistent with our findings that three doses of either BNT162b2 or CoronaVac had relatively high effectiveness (97.9%) against severe infections compared to two doses during the Omicron BA.2 period [25]. In Shanghai, China, Huang et al. observed a low protection of inactivated vaccine against Omicron BA.2 infection with VE of 17.9% for three doses or 15.9% for two doses compared to unvaccinated [53]. In Qatar, it was estimated that the effectiveness of the third dose of BNT162b2 vaccine was 43.7% (95% CI 36.5, 50.0) against Omicron BA.2 compared to unvaccinated in the first month [21], which outperformed two doses, but a decline in VE during the following weeks was also observed. In the UK, two doses of BNT162b2 or ChAdOx1-S-(AZD1222) was estimated to provide less than 20% protection in preventing symptomatic Omicron infection, whereas three doses increased this protection to 55% to 80% [54].

Although the booster dose of inactivated vaccines provided strong initial protection against SARS-CoV-2 infection, the protection may also wane 6 months after vaccination. Our finding has been expounded by previous literature, showing that noteworthy effectiveness against infection by Omicron BA.1 was observed in the first 2 months after booster vaccination (72.1%) compared to unvaccinated, followed by a significant decline to 51.2% [55], and Wang et al. also observed receiving dose 3 mRNA vaccines less than 180 days ago lowered this risk (compared to the unvaccinated) by a factor of 46.3 and 19.2 for the delta and omicron variants, respectively [56]. Few studies have focused on the effectiveness of the third dose of inactivated vaccine against Omicron infection regardless of symptom status. The effectiveness of the Sinovac (i.e., CoronaVac) booster vaccine against symptomatic disease was found to significantly decrease from 15.0% in the first 2 months to 0.4% during the third month and the sixth month, while the effectiveness against severe disease decreased from 71.3 to 65.4% [57]. A large population-based case–control study in the Netherlands showed a significant decrease in the protective effect against BA.1 and BA.2 after the third dose of vaccine [58]. Additionally, data from the US CDC showed that there were only 19% effectiveness of the third dose against the Omicron variant after 5 months [39]. Therefore, our research was aimed at a supplementary target to fill the gaps in the knowledge of inactivated booster marginal effectiveness against infection by Omicron BA.5.

We performed various subgroup analyses for 3-dose inactivated vaccine in different age. We report an evidence for protection among the young adults, but such evidence was not detected among older age groups. Until August 2022, the full vaccination coverage for elderly individuals aged over 60 years was 85.6% in China, while the booster vaccination coverage for the elderly was 67.8%, which was yet not reach the coverage in the US (92.1% and 70.7%) and Japan (92.4% and 90.3%) [59, 60]. Furthermore, it has already been highlighted that elderly individuals suffer from a higher risk of infection or developing severe clinical conditions of COVID-19, and the VE among the elderly is also far less than that for young people [61–63].

We reported that the VE was high when both infected individuals and their contacts were vaccinated with three doses. This finding was compatible with the first point of our findings that the overall effectiveness of booster vaccination against Omicron variant infection outperformed that of the primary series of two-dose vaccination. Unlike other places outside mainland China, quarantine and lockdown have been implemented for a longer time as a pandemic intervention strategy in the Xinjiang Uygur Autonomous Region; thus, the household contact setting accounted for a larger proportion of the transmission of COVID-19. In the subgroup analysis based on the matched cohort study, the cumulative incidence among those who received three doses of vaccine was much lower than that among those who received two doses of vaccine among the household contact subgroups after city lockdown and 15 to 180 days from the last dose. The booster vaccine was effective in preventing infection among households and after the lockdown, but this protective effect was not significant in non-household contact settings or before the lockdown, which may result from the wide range of close contact tracing. After 180 days, the protection of primary two-dose vaccines was no longer significant, indicating the demand for booster vaccination and consistent with the strategies in Hong Kong [64], and the US [65]. The difference in the vaccination status of the source cases (i.e., with 3 doses versus 0–2 doses) was statistically insignificant. However, an additional comparison between the blue curves in Fig. 3E, F shows that the cumulative incidences seeded by source cases who had received 0–2 doses were different from those seeded by source cases who had received 3 doses of vaccine (*p*-value < 0.001 from log-rank test). This implies that 3-dose vaccination might also contribute to reducing the risk of transmission, but further investigation is needed.

There were several limitations in this study. First, we excluded participants with ages below 18 years because the vaccination policies were different for minors from adults in mainland China. Therefore, the distributions of baseline conditions as well as vaccine dosage among adults and minors were unbalanced for comparison. Second, at the “post-pandemic” stage of COVID-19, it would be more informative to assess VE against clinical manifestations, duration of SARS-CoV-2 infection, and the risk of hospitalization. However, there was no information on the clinical severity recorded in our dataset, and thus, our findings on VE cannot be extended to the more severe clinical spectrum of COVID-19. Third, since no large-scale outbreak occurred in Urumqi before our study period owing to the “zero-COVID” policy in China, the scenario of reinfection was neglected. In addition, since this study is not based on a community-level seroprevalence study, we cannot overrule the chances that even a strict surveillance program might miss some cases, which could lead to inaccurate VE estimates. We presume this probable issue has a minor impact on our main conclusion as both test-positive and test-negative contacts may be equally likely to be identified in a non-symptom-based contact-tracing program. Fourth, the quantity of viral load for the source cases at the time of exposure and the duration and mode of exposure (e.g., talk, shared room) could be potential confounders in our study, which were not considered in the analysis due to limited access to data. Nonetheless, because city-wide mass testing was imposed daily for all residents since the beginning of the local outbreak, and all test-positive cases and their close contacts were quarantined immediately, all transmission events were believed to occur at a very early stage of the disease for the source cases, such that the difference in viral load expected to be minimal. Moreover, by introducing the types of contact settings (household or non-household) we partially accounted for the effect of exposure mode. Finally, our results should be interpreted in the context of strict “zero COVID-19 policy”, under which most close contact only experienced limited level of exposure. With repeated or high-level exposure to SARS-CoV-2, we suspected that a relatively large fraction of close contacts may be infected during the course of COVID-19 epidemic.

## Research in context

Evidence before this study: with the rapid evolution of SARS-CoV-2, previous understanding of the protective performance of COVID-19 vaccines against the emerging genetic variants of SARS-CoV-2 (e.g., Omicron) is becoming increasingly important, especially for individuals with asymptomatic infection, because of their potentials for seeding downstream secondary transmission and for developing herd immunity. The assessment of vaccine effectiveness (VE) against asymptomatic Omicron infections generally lacking because the ascertainment of asymptomatic or mild infections was potentially challenging, despite the majority of Omicron infections being asymptomatic. Thus, VE estimates under common study designs, including test-negative designs, could bias toward more severe clinical conditions or subgroups of populations with high test-seeking behaviors. As of December 2022, we found 1 peer-reviewed cohort study based on the population in Hong Kong, China that assessed the VE of BNT162b2 and CoronaVac against asymptomatic Omicron BA.2 infection using real-world individual-level data. Owning to the previous “zero-COVID” policy in mainland China, COVID-19 was at a relatively low level before December 2022, and thus no VE estimate of BBIBP-CorV against Omicron infection in mainland China was published. To our knowledge, to date (December 2022), no study has reported the VE of BBIBP-CorV booster against Omicron BA.5 asymptomatic infection.

Added value of this study: in an Omicron BA.5 seeded outbreak in Urumqi, the capital and largest city in Xinjiang Uygur Autonomous Region of China, we identified 37,628 adult close contacts of COVID-19 with 2 or 3 doses of inactivated vaccines before exposure from August 1 to September 7, 2022. After matching for baseline conditions, we assessed the effectiveness of inactivated COVID-19 vaccines (mainly BBIBP-CorV) against Omicron infection, regardless of symptoms, in a real-world setting. The overall VE of booster dose versus 2-dose against Omicron BA.5 infection was 35.5% (95% CI 2.0, 57.5), with an effectiveness of 60.2% (95% CI 22.8, 79.5) for 15–180 days after vaccination, but decreased to 35.0% (95% CI 2.8, 56.5) after 180 days. These findings were the first VE estimates against SARS-CoV-2 Omicron BA.5 infection in mainland China.

Implications of all the available evidence: moderate but significant protective effects against asymptomatic and symptomatic Omicron BA.5 infection were found for the booster doses of inactivated vaccine. The VE estimates were important contributions to informing vaccination policy in places where vaccine coverage remains low or inactivated vaccines were in-use. Thus, it is important to assess the vaccine performance against emerging genetic variants of SARS-CoV-2, as they evolved, regardless of the background vaccine coverage.

## Data Availability

The original database containing confidential patient information cannot be made publicly available. The anonymized data used in this study were available based on reasonable request to the corresponding authors.
